# Kinetic profiling of novel spirobenzo-oxazinepiperidinone derivatives as equilibrative nucleoside transporter 1 inhibitors

**DOI:** 10.1007/s11302-023-09948-9

**Published:** 2023-07-10

**Authors:** Anna Vlachodimou, Jara Bouma, Michel De Cleyn, Didier Berthelot, Stefan Pype, Jean-Paul Bosmans, Herman van Vlijmen, Berthold Wroblowski, Laura H. Heitman, Adriaan P. IJzerman

**Affiliations:** 1 Division of Drug Discovery and Safety, Leiden Academic Centre for Drug Research (LACDR), Leiden University, P.O. Box 9502, 2300 RA Leiden, The Netherlands; 2 Janssen Research and Development, Antwerpseweg 30, 2340 Beerse, Belgium

**Keywords:** Target binding kinetics, (Human) nucleoside transport protein (hENT1, SLC29A1), Radioligand binding experiments, Transport inhibitors, Adenosine

## Abstract

Evaluation of kinetic parameters of drug-target binding, *k*_*on*_, *k*_*off*_, and residence time (RT), in addition to the traditional in vitro parameter of affinity is receiving increasing attention in the early stages of drug discovery. Target binding kinetics emerges as a meaningful concept for the evaluation of a ligand’s duration of action and more generally drug efficacy and safety. We report the biological evaluation of a novel series of spirobenzo-oxazinepiperidinone derivatives as inhibitors of the human equilibrative nucleoside transporter 1 (hENT1, SLC29A1). The compounds were evaluated in radioligand binding experiments, *i.e.*, displacement, competition association, and washout assays, to evaluate their affinity and binding kinetic parameters. We also linked these pharmacological parameters to the compounds’ chemical characteristics, and learned that separate moieties of the molecules governed target affinity and binding kinetics. Among the 29 compounds tested, 28 stood out with high affinity and a long residence time of 87 min. These findings reveal the importance of supplementing affinity data with binding kinetics at transport proteins such as hENT1.

**Supplementary Information:**

The online version contains supplementary material available at 10.1007/s11302-023-09948-9.

## Introduction

Nucleosides are critical endogenous molecules that are formed by fusion of pyrimidine or purine bases with either a ribose or deoxyribose sugar moiety. Subsequent phosphorylation converts the molecules into nucleotides. Both nucleosides and nucleotides serve as metabolic precursors in the synthesis of nucleic acids, play a crucial role in energy metabolism, and are involved in signaling pathways and enzyme regulation and metabolism [1, 2]. Due to the important physiological role of nucleosides, nucleoside analogs have been introduced as therapeutic molecules. They have been and are a core strategy in the treatment of cancer and viral infections [3].

The translocation of nucleosides and nucleoside analogs inside and outside of cell membranes occurs via two nucleoside transporter (NT) families: the concentrative (CNTs; SLC28) and the equilibrative NTs (ENTs; SLC29). In addition, NTs are involved in the recycling of nucleosides in cells lacking de novo synthesis, such as erythrocytes [4]. Human ENT1 (hENT1; SLC29A1) is the best-studied ENT and the major plasma membrane nucleoside transporter, highly expressed in tissues such as erythrocytes, vascular endothelium, and the gastrointestinal tract [2, 5, 6]. It brings its substrates over the plasma membrane in a bidirectional, sodium-independent manner by following the concentration gradient in order to establish equilibrium [7, 8]. Moreover, it has recently been shown that hENT1 is essential for erythropoiesis, the development of erythrocytes from hematopoietic stem cells [9]. In obesity, yet another recent finding, ENT1 is an important target too with an intriguing role for the nucleoside inosine [10, 11].

**Fig. 1:**
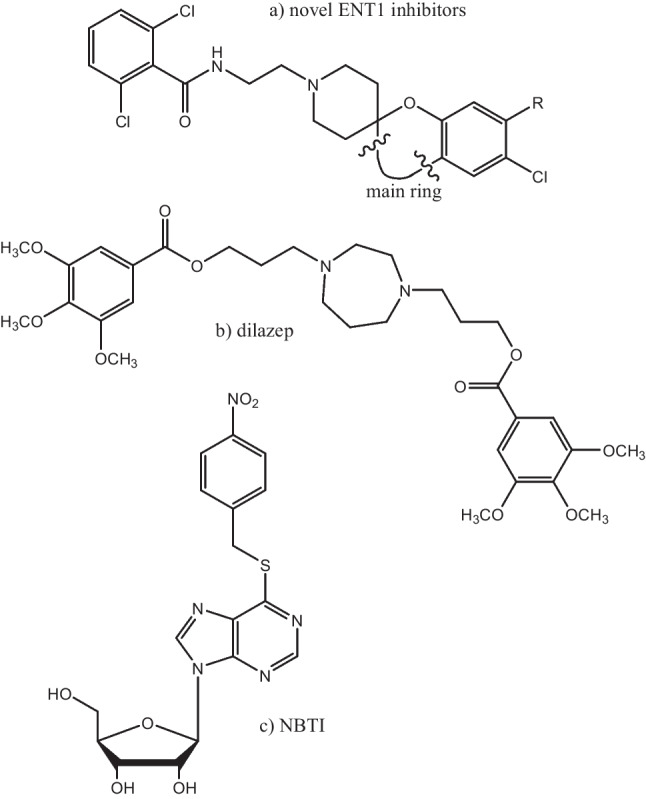
Chemical structures of hENT1 inhibitors. **a** Generalized chemical structure of novel ENT1 inhibitors with a diversified “main ring” (as in Table 5) and typical R substituents (R^1^-R.^4^, as in Tables 1, 2, 3 and 4). **b** Dilazep. **c** 4-nitrobenzylthioinosine (NBTI)

Pharmacological inhibition of hENT1 is a promising therapeutic strategy for many diseases. Due to its increased expression in a variety of cancers, *e.g.*, breast cancer [12] and pancreatic adenocarcinoma [13], the use of hENT1 inhibitors presents a potential anti-cancer therapy [14]. As an alternative and add-on cancer therapy, the administration of hENT1 inhibitors in combination with anti-cancer nucleoside drugs transported by other NTs has been proposed. Such combination therapy would enhance the effect of nucleoside drugs by preventing cellular efflux, as well as reduce the occurrence of drug resistance [7, 15]. Lastly, hENT1 inhibition has therapeutic benefits by the modulation of the extracellular concentration of adenosine [5]. Clinical trials with draflazine, an hENT1 inhibitor, showed promising results in patients with unstable coronary disease, by enhancing the anti-ischemic and cardioprotective effects of endogenous adenosine [16]. Two potent hENT1 inhibitors, dilazep (Fig. 1) and dipyridamole, are already on the market as vasodilators [17, 18], but the development of novel inhibitors with improved efficacy and selectivity is desired (2).

**Table 1:**
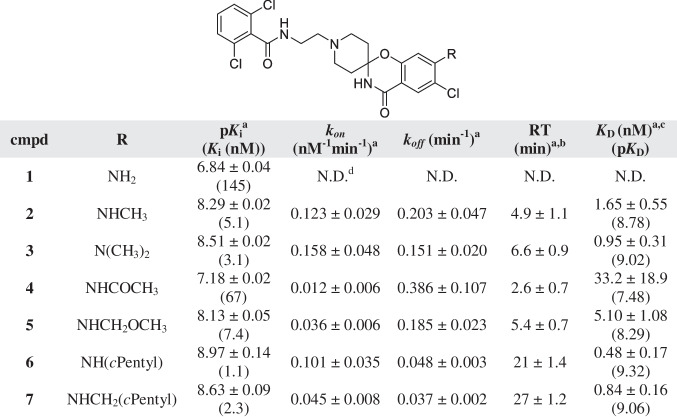
Affinity (pK_*i*_) and kinetic parameters (*k*_*on*_, *k*_*off*_, RT) of hENT1 inhibitors 1–7

^a^Values represent the mean ± SEM of at least three individual experiments, performed in duplicate

^b^RT = 1/*k*_*off*_

^c^Kinetic *K*_D_ values, defined as *K*_D_ = *k*_*off/*_*/k*_*on*_*.*

^d^N.D. = not determined

**Table 2:**
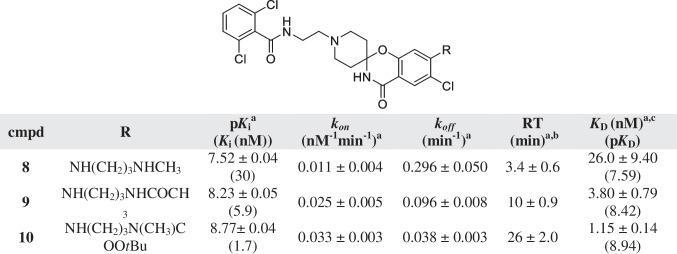
Affinity (pK_*i*_) and kinetic parameters (*k*_*on*_, *k*_*off*_, RT) of hENT1 inhibitors 8–10

^a^Values represent the mean ± SEM of at least three individual experiments, performed in duplicate

^b^RT = 1/*k*_*off*_

^c^Kinetic *K*_D_ values, defined as *K*_D_ = *k*_*off/*_*/k*_*on*_

**Table 3:**
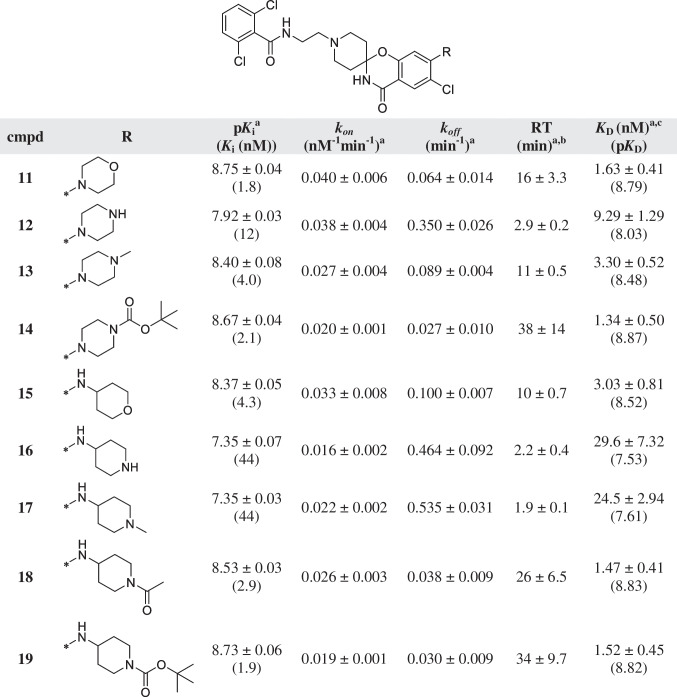
Affinity (pK_*i*_) and kinetic parameters (*k*_*on*_, *k*_*off*_, RT) of hENT1 inhibitors 11–19

^a^Values represent the mean ± SEM of at least three individual experiments, performed in duplicate

^b^RT = 1/*k*_*off*_*.*

^c^Kinetic *K*_D_ values, defined as *K*_D_ = *k*_*off/*_*/k*_*on*_

**Table 4:**
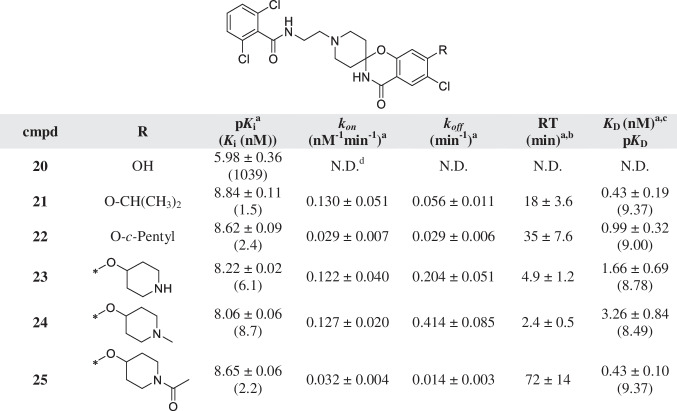
Affinity (pK_*i*_) and kinetic parameters (*k*_*on*_, *k*_*off*_, RT) of hENT1 inhibitors 20–25

^a^Values represent the mean ± SEM of at least three individual experiments, performed in duplicate

^b^RT = 1/*k*_*off*_

^c^Kinetic *K*_D_ values, defined as *K*_D_ = *k*_*off/*_*/k*_*on*_

^d^N.D. = not determined

It is increasingly realized that ligand selection based solely on affinity, an equilibrium parameter, is not necessarily a good predictor for in vivo efficacy. Contrarily, the study of target binding kinetics, *i.e.*, association, *k*_*on*_, and dissociation, *k*_*off*_, rate constants as well as residence time (RT), on a majority of targets, including enzymes and G protein-coupled receptors (GPCRs), has provided emerging evidence that in vivo efficacy is often linked to optimized binding kinetic parameters of the ligand [19–21]. Although the binding kinetics of some hENT1 inhibitors have been studied 2, their incorporation in drug discovery efforts,* i.e.*, by delineating structure-kinetic relationships (SKR) next to the structure-affinity relationships (SAR), has been limited [22].

In this study, we report the biological evaluation of a novel series of spirobenzo-oxazinepiperidinone derivatives as hENT1 inhibitors with a structure different to the marketed products dilazep and dipyridamole. These compounds were evaluated in radioligand displacement assays to determine their affinity and in radioligand competition association and washout assays that enabled their kinetic characterization. We learned that both the speed of target engagement and the dissociation of the inhibitor-transporter complex play a distinctive role in the compounds’ affinities.

## Materials and methods

### Chemistry

All new hENT1 inhibitors were synthesized at Janssen Pharmaceutica (Beerse, Belgium), and checked for identity and purity. The general procedure for the preparation of these compounds has been described [23]. In it are the general synthetic schemes (pp. 6–9), the detailed synthetic procedures of 57 intermediates (pp. 16–62), and 33 final compounds (pp. 63–77). The identity and purity of the final compounds were characterized by their melting points (table F-6) and various LC/MS procedures with their respective retention times and exact mass values (table F-7).

### Biology

#### Chemicals and reagents

Bovine serum albumin (BSA) and the bicinchoninic acid (BCA) protein assay kit were purchased from Fisher Scientific (Hampton, New Hampshire, USA). [^3^H]NBTI (specific activity 33.1 Ci mmol^−1^) was purchased from PerkinElmer (Groningen, The Netherlands) and NBTI (4-nitrobenzylthioinosine) was obtained from Sigma-Aldrich (Steinheim, Germany). Erythrocytes were obtained from Sanquin (Amsterdam, The Netherlands). All other chemicals were purchased from standard commercial sources.

#### Membrane preparation

Erythrocyte membrane preparation was performed as previously described [22]. Briefly, erythrocytes were stirred in lysis buffer. After homogenization and centrifugation, the resulting membrane pellets were collected and washed multiple times until the supernatant was colorless. Then, the pellets were homogenized in storage buffer, divided in aliquots, and stored at − 80 °C. Membrane protein concentrations were measured using the biconchoninic acid (BCA) method [24].

#### Radioligand binding assays

Membranes were thawed and homogenized using an Ultra Turrax homogenizer at 24,000 rpm. Assay buffer (50 mM Tris–HCl pH 7.4, 0.1% (w/v) 3-[(3-cholamidopropyl)dimethylammonio]-1-propanesulfonate hydrate (CHAPS)) was used to dilute the samples to a total reaction volume of 100 μL (except for the washout assay where it was 400 μL) containing 1 μg membrane protein and 4 nM [^3^H]NBTI. Assay buffer, (radio)ligands and membranes were cooled at 10 °C prior to the experiment. Incubations were performed at 10 °C. Nonspecific binding was determined in the presence of 10 μM NBTI. In all cases, dimethyl sulfoxide (DMSO) concentrations were kept ≤ 0.25% and total binding did not exceed 10% of the [^3^H]NBTI present in the assay in order to prevent ligand depletion.

All radioligand binding assays were performed as previously described [22]. In short, displacement experiments were performed using [^3^H]NBTI (*K*_D_ = 1.1 nM 22) and a competing unlabeled ligand at multiple concentrations ranging from 0.1 nM to 10 µM. Sample incubation lasted for 1 h. Competition association experiments were carried out by incubation of [^3^H]NBTI and a competing ligand at its IC_50_ concentration. The amount of transporter-bound radioligand was determined at different time points up to 1 h. All incubations were terminated by rapid vacuum filtration over 96-well Whatman GF/C filter plates using a PerkinElmer Filtermate harvester (PerkinElmer, Groningen, The Netherlands). Subsequently, filters were washed ten times using ice-cold wash buffer (50 mM Tris–HCl, pH 7.4) and filter-bound radioactivity was determined by liquid scintillation spectrometry using a 2450 Microbeta^2^ scintillation counter (PerkinElmer).

Washout experiments were performed by the incubation of inhibitors **18**, **23**, and **25** at a concentration of 10 × IC_50_ with erythrocyte membranes at 10 °C for 1 h while shaking at 1000 rpm. Centrifugation at 13,200 rpm (16,100 × g) at 4 °C for 5 min separated pellet and supernatant, with the latter containing the unbound ligand being removed. Pellets were resuspended in 1 mL of assay buffer, and samples were incubated for 10 min at 10 °C. In total, four centrifugation-washing cycles were performed. The pellet-membranes were resuspended in a total volume of 400 μL containing 4 nM [^3^H]NBTI and were incubated at 10 °C for 1 h. Rapid filtration through GF/C filters using a Brandel harvester (Brandel, Gaithersburg, MD) terminated the experiment. Filters were washed three times using ice-cold wash buffer and the samples were counted by scintillation spectrometry using a Tri-carb 2900 TR liquid scintillation counter (Perkin Elmer, Boston, MA).

#### Data analysis

Data analyses were performed using the GraphPad Prism 7.0 software (GraphPad Software Inc., San Diego, CA, USA). For displacement assays, pIC_50_ values were obtained by nonlinear regression curve fitting to a sigmoidal concentration–response curve using the equation: *Y* = bottom + (top–bottom)/(1 + 10^(*X*-LogIC_50_)). pK_*i*_ values were converted from pIC_50_ and the saturation *K*_D_ values using the Cheng-Prusoff Eq. 25: *K*_*i*_ = IC_50_/(1 + [radioligand]/*K*_*D*_). Association and dissociation rate constants for unlabeled ENT1 inhibitors were determined by nonlinear regression analysis of competition association data as described by Motulsky and Mahan [25].

$$\begin{array}{c}{K}_{A}={k}_{1}[L]\cdot {10}^{-9}+{k}_{2}\\ {K}_{B}={k}_{3}[I]\cdot {10}^{-9}+{k}_{4}\\ S=\sqrt{{({K}_{A}-{K}_{B})}^{2}+4\cdot {k}_{1}\cdot {k}_{3}\cdot L\cdot I\cdot {10}^{-18}}\\ {K}_{F}=0.5({K}_{A}+{K}_{B}+S)\\ {K}_{S}=0.5({K}_{A}+{K}_{B}-S)\\ Q=\frac{{B}_{\mathrm{max}}\cdot {k}_{1}\cdot L\cdot {10}^{-9}}{{K}_{F}-{K}_{S}}\\ Y=Q\cdot (\frac{{k}_{4}\cdot ({K}_{F}-{K}_{S})}{{K}_{F}\cdot {K}_{S}}+\frac{{k}_{4}-{K}_{F}}{{K}_{F}}{e}^{(-{K}_{F}\cdot X)}-\frac{{k}_{4}-{K}_{S}}{{K}_{S}}{e}^{(-{K}_{S}\cdot X)})\end{array}$$

where *k*_1_ and *k*_2_ are the *k*_*on*_ (M^−1^ min^−1^) and *k*_*off*_ (min^−1^) of [^3^H]NBTI, respectively, *L* is the radioligand concentration (nM), *I* is the concentration of unlabeled competitor (nM), *Y* is the specific binding of the radioligand (disintegrations per minute, DPM), and *X* is the time (min). Fixing these parameters with the help of a control curve, where no unlabeled compound was used, allows the calculation of the following parameters: *k*_3_ (M^−1^ min^−1^), which is the *k*_*on*_ value of the unlabeled ligand; *k*_4_ (min^−1^), which is the *k*_*off*_ value of the unlabeled ligand, and *B*_max_ that equals the total binding (DPM). All competition association data were globally fitted. The residence time (RT) was calculated using RT = 1/*k*_*off*_ [26]. The kinetic affinity *K*_D_ was calculated by association and dissociation rates using the following equation:

$${K}_{D}={k}_{off}/{k}_{on}$$

All values are shown as mean ± SEM of at least three independent experiments performed in duplicate. Statistical analysis was performed if indicated, using a one-way ANOVA with Dunnett’s post-test (^###^*P* < 0.001; ^##^*P* < 0.01; ^#^*P* < 0.05) or an unpaired Student’s *t* test (***P < 0.001; **P < 0.01; *P < 0.05). Observed differences were considered statistically significant if *P*-values were below 0.05.

## Results

### Radioligand binding assays to determine affinity and target binding kinetics

**Fig. 2:**
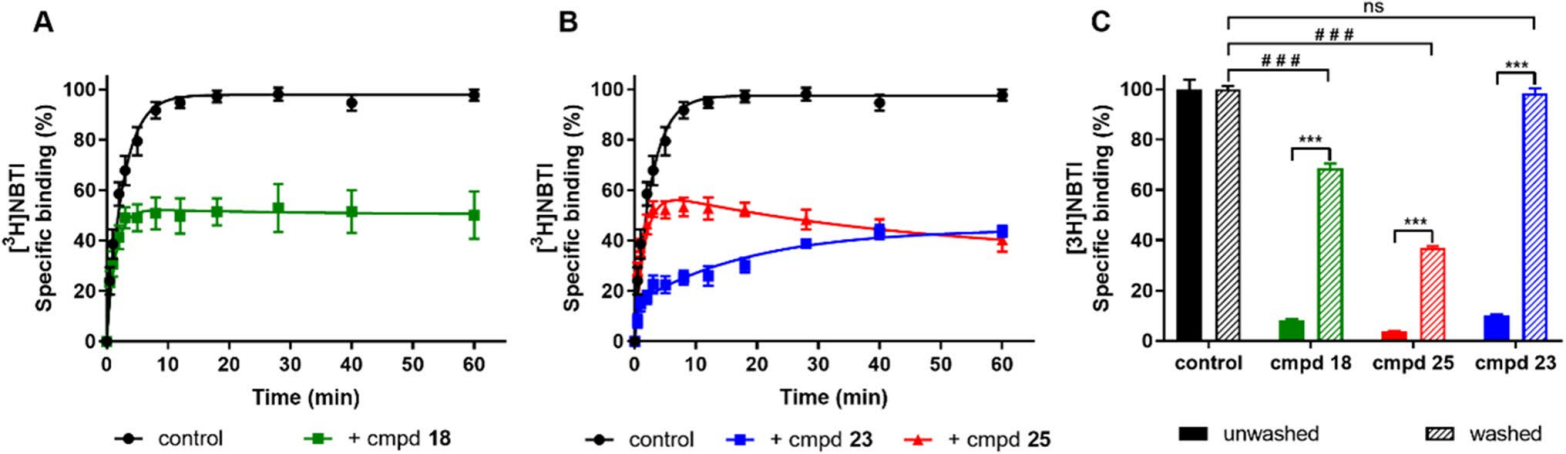
Competition association of specific [^3^H]NBTI binding to hENT1 on erythrocyte membranes (10 °C) in the absence or presence of IC_50_ concentration of a similar (**A**), shorter and longer (**B**) RT compound (cmpd) compared to the control. Ligand binding of compounds at 10 × IC_50_ concentration before and after washing step, compared to the control radioligand binding without the presence of any competitor (**C**). Data are shown as mean ± SEM from at least three independent experiments performed in duplicate. *** *p* ≤ 0.0001 determined in an unpaired *t* test with Welch’s correction. ^# # #^ *p* ≤ 0.0001 determined in a one-way ANOVA test with Dunnett’s correction

The binding affinity of all compounds was determined on human erythrocyte membranes at 10 °C in the presence of 4 nM of the tritium-labeled hENT1 inhibitor NBTI ([^3^H]NBTI). The fast dissociation of the radioligand prevented performing kinetic experiments at higher temperature. All compounds were found to inhibit specific radioligand binding to the hENT1 transporter and the determined affinities are listed in Tables 1, 2, 3, 4 and 5. The compounds had moderate to high affinity for the transporter ranging from 1039 nM for compound **20** to 0.58 nM for compound **29** (Tables 4 and 5). Subsequently, all compounds with affinity values lower than 100 nM [2–19, 21–24, 27] were evaluated in a radioligand competition association assay, to determine the kinetic parameters *k*_*on*_ and *k*_*off*_. This assay is based on the Motulsky and Mahan model and characterizes the time-dependent binding of two competing ligands on the same target binding site [25]. For the purposes of the assay, the specific binding of [^3^H]NBTI was measured at different time points during an incubation of 60 min in the absence and presence of an IC_50_ concentration of the competing inhibitor. Figure 2 A and B present the curves from inhibitors **18**, **23**, and **25** with similar, shorter, and longer RTs compared to the radioligand used, respectively. A longer RT compound presents a characteristic overshoot followed by a steady decrease in specific radioligand binding, which eventually reaches equilibrium around 50%. A compound with similar RT as the radioligand, between 20 and 30 min in this case, displays a curve with a similar shape to the control curve of [^3^H]NBTI (RT ~ 27 min), while a shallow, slowly ascending curve is typical for compounds with a shorter RT.

**Table 5:**
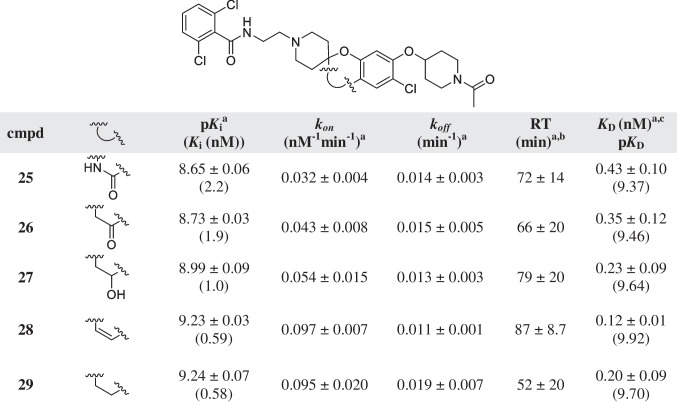
Affinity (pK_*i*_) and kinetic parameters (*k*_*on*_, *k*_*off*_, RT) of hENT1 inhibitors **25**–**29** with modifications on the main ring

^a^Values represent the mean ± SEM of at least three individual experiments, performed in duplicate

^b^RT = 1/*k*_*off*_

^c^Kinetic *K* values, defined as *K*_D_ = *k*_*off/*_*/k*_*on*_

#### Structure-affinity relationships (SAR) and structure-kinetic relationships (SKR)

Substitution on the 4-position of the phenyl ring (compounds **1–25**). The obtained affinities (*K*_i_ values) and kinetic profiles (*k*_*on*_, *k*_*off*_ values and RTs) were used to derive SAR and SKR of the hENT1 inhibitors. In this series of compounds, phenyl ring analysis consisted of an extensive substitution on the 4-position by different side chains, represented by R groups (Tables 1, 2 and 3).

Substitution of a primary amine (**1**) to the phenyl ring resulted in a modest affinity of 145 nM and therefore the RT was not determined. Introduction of a secondary or tertiary amine by substitution of one (**2**) or two (**3**) methyl groups resulted in an approximately 30-fold increase in affinity compared to **1**, and a short RT for both compounds. Addition of an acetamide to the phenyl ring (**4**) led to a reduced affinity compared to **2** and **3**, while RT remained short. Association rate constant *k*_*on*_ was decreased by tenfold and 13-fold vs. **2** and **3**, respectively. Addition of a methoxymethyl (**5**) increased both the affinity and RT compared to **4**, to values similar to the ones of secondary and tertiary amine substitution. The greatest increase in both affinity and RT was obtained when substituting a cyclopentyl group, either directly (**6**) or with an additional carbon (**7**) to the secondary amine. Changes in *k*_*on*_ were also observed, with **7** presenting a twofold decrease compared to** 6**.

As the longer bulkier side chains of **6** and **7** resulted in an increased RT, longer amine substituents were introduced. A propylamine linker was added to the aromatic amine and its substitution yielded compounds **8** to **10** (Table 2). Compound **8** consisted of a secondary amine with a methyl group as substituent and its affinity was 30 nM, while the RT was short (3.4 min). Further substitution of the propylamine linker (**9, 10**) led to an increased affinity and RT compared to inhibitor **8**. Specifically, the addition of a *tert*-butyloxycarbonyl (BOC) group and a methyl to the amine (**10**) led to an affinity of less than 2 nM and an RT of 26 min, comparable to inhibitors **6** and **7**. All in all, the increased substituent size of compounds **8** to **10** led to an increased affinity, in addition to an increased association and decreased dissociation rate constant.

A combination of the cyclic characteristics of compounds **6** and **7** with the propylamine linker of compounds **8** to **10** that exhibited an increase in affinity and RT led to the design and synthesis of inhibitors **11** to **19** (Table 3). The morpholino group of compound **11** and the *para*-substituted piperazines of **12** to **14** resulted in similar *k*_*on*_ values, whereas *k*_*off*_ values and as a consequence RT varied up to 13-fold, with the substituted BOC-group (**14**) yielding the longest RT of all four compounds. For compounds **15** to **19**, the aromatic amine was substituted with tetrahydropyran (**15**) or a substituted piperidine (**16** to **19**) and therefore, the functional groups are located four positions away from the amide, comparable to the inhibitors with a propyl linker. Similarly as for inhibitors **12** and **13**, the compounds with a secondary (**16**) or tertiary (**17**) amine in the ring showed a relatively modest affinity and a fast dissociation from the target. The *N*-acetyl (**18**) and the *N*-BOC-group (**19**) both yielded a high affinity and a long RT, comparable to **14**.

In addition to the results obtained with the subseries of the amino-substituted phenyl ring, an oxygen linker was introduced, leading to inhibitors **20** to **25** (Table 4). Introducing a hydroxyl group (**20)** resulted in a significantly decreased affinity (*K*_i_ = 1039 nM) and therefore the binding kinetics were not measured. Substitution of the phenyl’s 4-position with an ether with bigger nonpolar groups (**21, 22**) increased the affinity to the nanomolar range. The binding kinetics for isopropyl-substituted **21** were quite fast for both the association and dissociation to and from the target. In contrast, cyclopentyl-substituted **22** presented a slower association and dissociation rate constant compared to **21**. In addition, an ether linkage was used to connect the main scaffold with *para*-substituted piperidines. The piperidine (**23**) and *N*-methylpiperidine (**24**) derivatives showed similar association rate constants and a fast dissociation of less than 5 min. The *N*-acetylpiperidine (**25**) analog yielded both a slow association and dissociation rate, resulting in an RT of 72 min, the longest in this series.

Substitution in the main ring (compounds **25**–**29**).

After the exploration of the “right-hand” side of the scaffold, changes on the main ring were introduced in order to evaluate their significance in the SAR and SKR. As the *N*-acetylpiperidine ether at the 4-position of the phenyl ring (**25**) caused the longest RT as well as a high affinity, it was maintained as the R-substitution, while alterations were performed to the main ring (Table 5). A reduction of polarity of the ring (**26-29**) led to an increased affinity as well as an increase in association rate constant. However, the RTs stayed within a similar long range as **25**, where compound **28** had the longest RT of 87 min.

### Washout assay

A washout assay was used to validate the findings in the competition association assays (Fig. 2C). Three chemically related inhibitors differing only at the 4-phenyl substituent yet with distinct RTs were examined, *i.e.*, compound **25** presenting a long RT, compound **18** with a medium RT (similar to the radioligand) as well as compound **23** that displayed a short RT. Following a 1h pre-incubation with a 10 × IC_50_ concentration of compound and four subsequent wash and centrifugation cycles, [^3^H]NBTI was co-incubated and radioligand binding was determined. The unwashed condition was also similarly assessed, but no wash and centrifugation cycle occurred before the determination of radioligand binding. The results of both washed and unwashed samples were compared to the control condition without any competitor (100% washed and unwashed radioligand binding, respectively).

All inhibitors showed a significant increase in [^3^H]NBTI binding after four extensive washing steps, suggesting all inhibitors were washed away to some extent due to dissociation from the target. The recovery of [^3^H]NBTI binding increased in relation to the inhibitor’s duration of binding to the target (Fig. 2C). Long RT inhibitor **25** was washed away only by 37% after four extensive washing steps, suggesting that more than 60% of this inhibitor was still bound to the target. On the contrary, short RT inhibitor **23** was completely washed away, as the radioligand was found to bind to all binding sites after the washing steps. Compound **18** displayed an intermediate behavior with approximately 60% being washed away.

### Kinetic map

**Fig. 3:**
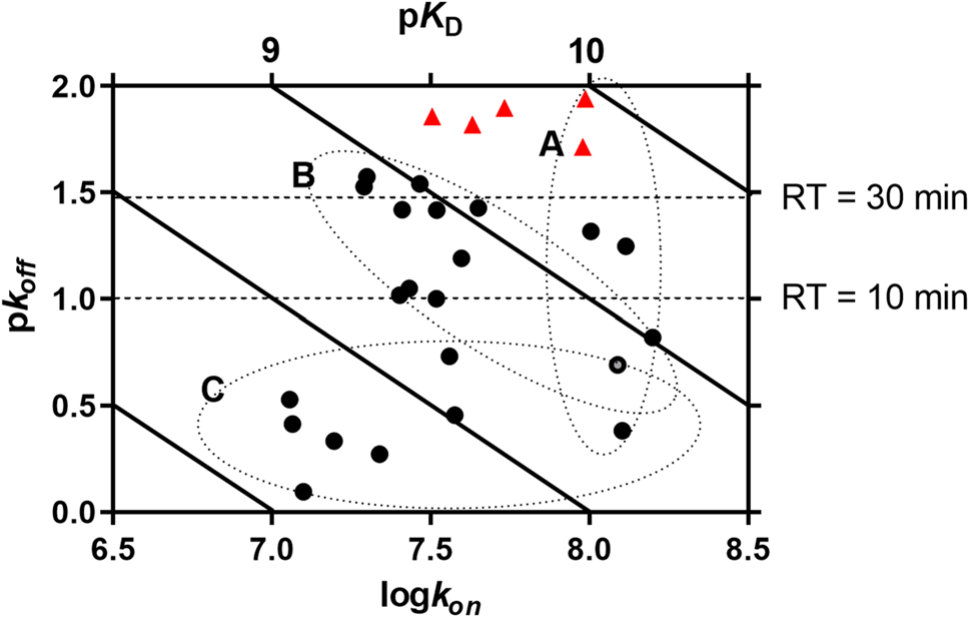
Kinetic map of all hENT1 inhibitors that were kinetically characterized through [^3^H]NBTI competition association assays. The kinetically derived affinity (*K*_*D*_) is represented by the diagonal parallel lines. The compounds are divided into short, medium, and long RT by the horizontal dashed lines, indicating an RT of 10 and 30 min. Group A: inhibitors that display similar *k*_*on*_ values but because of different *k*_*off*_ values have divergent *K*_*D*_ values. Group B: inhibitors with similar *K*_*D*_ values despite presenting diverse *k*_*off*_ and *k*_*on*_ values. Group C: inhibitors showing similar *k*_*off*_ values but due to differences in *k*_*on*_ have different *K*_*D*_ values. The red triangles correspond to inhibitors with modifications in the main ring

In an effort to obtain a better comparison of kinetic and affinity parameters, a kinetic map was created (Fig. 3).

In this map, the association (*x*-axis) together with the dissociation (*y*-axis) and the kinetic affinity (diagonal lines) values were plotted. Based on this representation, the compounds can be arbitrarily divided into three subgroups. Inhibitors that belong to group A (“vertical”) display similar association rate constants, but differ in their affinity for hENT1 due to diverse dissociation rate constants. Inhibitors in group B (“bisectorial”) have similar affinities but have many different combinations in association and dissociation rate constants. For example, inhibitors **3** and **22** have similar kinetic affinities (0.95 and 0.99 nM, respectively), yet their RTs are fivefold different with corresponding *k*_*off*_ values of 0.151 and 0.029 min^−1^, respectively. Lastly, group C (“horizontal”) represents inhibitors that exhibit similarly fast dissociation rate constants but differ in their affinity for hENT1 due to divergent association rate constants. The red triangles represent the inhibitors with modifications in the main ring. These follow a similar pattern as group C, where the association rate constants cause the difference in affinity. However, they are separated from group C by having a long RT over 30 min in contrast to less than 10 min for group C.

### Correlation plots

**Fig. 4:**
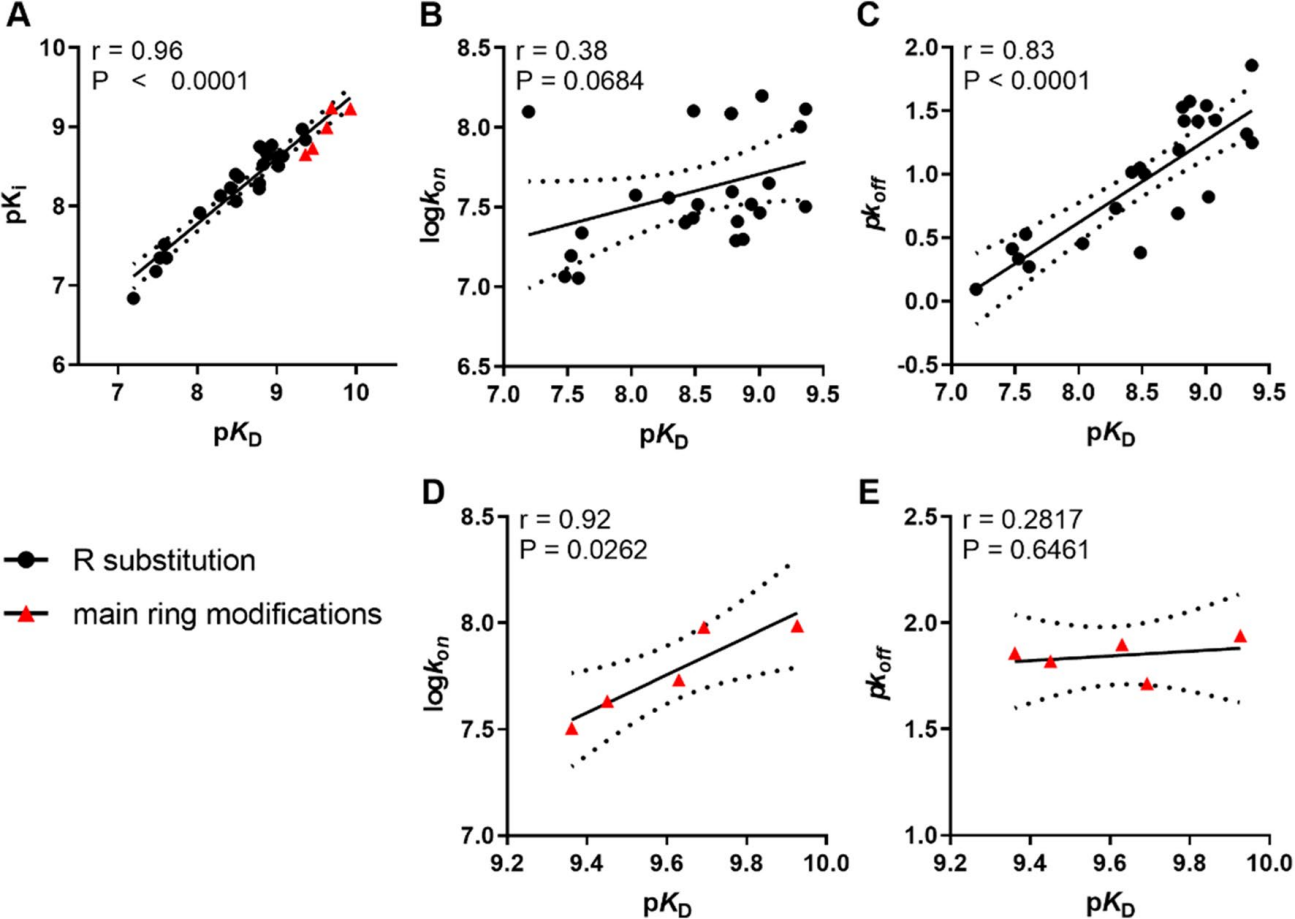
Correlation between affinity determined from typical displacement assays (pK_*i*_) and kinetic affinity determined based on parameters *k*_*on*_ and *k*_*off*_ obtained from competition association assays (pK_D_) (inhibitors **1**–**29**) (**A**); affinity (pK_D_) and association rate constant (log*k*_*on*_) of compounds sharing the same scaffold and different R substituents (inhibitors **1**–**25**) (**B**); affinity (pK_D_) and dissociation rate constant (pk_*off*_) of compounds sharing the same scaffold and different R substituents (inhibitors **1**–**25**) (**C**); affinity (pK_D_) and association rate constant (log*k*_*on*_) of compounds with modifications in the main ring (inhibitors **25**–**29**) (**D**); affinity (pK_D_) and dissociation rate constant (pk_*off*_) of compounds with modifications in the main ring (inhibitors **25**–**29**) (**E**). The solid line corresponds to the linear regression of the data and the dotted lines represent the 95% confidence intervals for regression. Inhibitors with modifications in the main ring are represented by red triangles and inhibitors with R substituents by the black dots. Data used in the plots are detailed in Tables 1, 2, 3, 4 and 5. Data are expressed as mean from at least three independent experiments performed in duplicate

To gain further insight into the relationship between the pharmacological parameters, correlation plots were constructed (Fig. 4).

The affinity obtained from the typical radioligand displacement assay (pK_i_) and the one calculated from the kinetic parameters (pK_D_) obtained from the radioligand competition association assay were found to be significantly correlated (Fig. 4A), validating the use of the competition association assay. In addition, the association rate constants (log*k*_*on*_) of inhibitors **1**–**25** were plotted against their kinetic affinity (pK_D_) (Fig. 4B), which showed a low, nonsignificant correlation (*r* = 0.38, *P* = 0.068). On the contrary, plotting the dissociation rate constants (pk_*off*_) and the kinetic affinity (pK_D_) demonstrated a strong correlation (*r* = 0.83, *P* < 0.0001) (Fig. 4C). As far as the modifications in the main ring are concerned, the correlations are opposite to the aforementioned. Association rate constants (log*k*_*on*_) and affinity were strongly correlated for the inhibitors **25**–**29** (*r* = 0.92, *P* = 0.026) (Fig. 4D), while dissociation rate constants (pk_*off*_) and affinity appeared not correlated at all (Fig. 4E).

Possible correlations to physicochemical properties of the phenyl’s *meta*-substituents (R) were also examined. The acid dissociation constants (pK_*a*_) as well as the distribution-coefficient (logD) at pH 7.4 of the substituents were first calculated and then used in the correlation analyses (SI: Figure S1 and Table S1). Affinity (pK_D_) and dissociation rate constant (pk_*off*_) appeared correlated (SI: Figure S2A, C) with pK_*a*_ (*r* =  − 0.657, *P* = 0.0012 and *r* =  − 0.695, *P* = 0.0003, respectively). As a result, all inhibitors with an easily protonated functional group R (**12, 13, 16, 17, 23, 24**) showed a lower affinity and shorter RT compared to the non-protonated functional groups (**11, 14, 15, 18, 19, 25**) at pH 7.4. Likewise, pK_D_ and pk_*off*_ were correlated with log (*r* = 0.636, *P* = 0.0008 and *r* = 0.655, *P* = 0.0004, respectively) providing evidence that the hydrophobicity of the R substituents plays a role in affinity and RT (SI: Figure S2D, F). No correlation (SI: Figure S2B, E) was found between the association rate constants (log*k*_*on*_) and pK_*a*_ (*r* =  − 0.247, *P* = 0.2798) or log*D* (*r* = 0.164, *P* = 0.4327).

## Discussion

Target binding kinetics has become an important parameter in early drug discovery, irrespective of the target class studied [28]. It helps in triaging and optimizing lead compounds with acceptable affinities for a given target, which adds to the armamentarium of the academic and industrial medicinal chemist [29]. This is beginning to be understood as well for an understudied class of potential drug targets, *i.e.*, transport proteins such as solute carriers (SLCs) [22]. In the latter study, a series of draflazine analogs was studied yielding their target binding kinetics for the human equilibrative nucleoside transporter 1 (hENT1 or SLC29A1). In the current study, we set out to characterize a new series of hENT1 inhibitors in a first but detailed set of experiments defining the compounds’ affinity and kinetic behavior at their target. We used radioligand binding studies for this purpose and, hence, actual blockade of nucleoside/nucleobase transport was not examined. Also, as mentioned in the “Results” section, we had to perform the studies at 10 °C rather than at physiological temperature. Higher temperatures would increase both association and dissociation rates. In terms of SKR, however, the ranking of compounds is believed to remain the same.

Many of the 29 compounds, represented by their general structure in Fig. 1, showed high affinity for hENT1. In case of a simple structure-affinity relationship (SAR) study, these inhibitors would have been considered similar and been treated equally in further compound selection. In fact, this is an issue in many drug discovery programs, in which medicinal chemists arrive at series of compounds with high but (seemingly) similar affinity. In addition, a simple SAR study may not necessarily result in “true” affinities for compounds with extreme binding kinetics. Compounds that occupy their target longer than the assay incubation time of *e.g.*, 30 or 60 min are not studied under equilibrium conditions, yielding affinity values that can significantly differ from their “true” equilibrium affinity. We took care to compare equilibrium (pK_*i*_) and kinetic affinity (p*K*_*D*_) data (Fig. 4A) and learned that in the present study these correlated well. This is indicative of the right assay conditions for the type of compounds evaluated in this study. Likewise, the target residence time (RT) in almost all cases was within the duration of the assay incubation time of 1 h.

It is often thought that the longer a drug occupies the target, the higher its affinity is. This is a misconception as is evident from Fig. 3. Compounds with the same/similar affinity (group B) may have widely variable *k*_*on*_ and *k*_*off*_ values. Also, compounds with similar *k*_*off*_ values (thus with a similar RT) can have very different affinities (group C). Therefore, equilibrium studies should best be enriched with a kinetics evaluation at the early stages of drug discovery to allow for a more thorough and complete classification of compounds, from which an informed follow-up is possible. As RT has been proven a predictive tool for in vivo efficacy, many kinetic studies have been directed towards optimization of dissociation rates [30]. However, attention has lately been given to the association rate constant as well, as it also plays a role in drug efficacy, due to possible increased rebinding to the target and/or increased drug-target selectivity [21, 31–33]. In addition, *k*_*on*_ has been described to be crucial for high receptor occupancy (“target engagement”) [31, 34, 35]. The latter may be relevant in view of the high local adenosine concentrations that are reached under *e.g.*, hypoxic conditions [36], competing for the binding site on hENT1. In the present series of compounds, the highest association rate constant was found in **3** (0.158 ± 0.048 nM^−1^ min^−1^), while the lowest was observed with **8** (0.011 ± 0.004 nM^−1^ min^−1^), an approx. 15-fold difference. However, it would require further in vivo studies to define whether a high *k*_*on*_ value in this series of compounds would favor drug efficacy. The target’s fate and lifetime are important too: a long RT for a quickly degraded target does not make much sense. In a recent study, the expression and functional activity of hENT1 was measured in primary hepatocytes isolated and cultured from four individual livers showing different levels of expression and activity after 4, 8, and 24 h [37]. This suggests that hENT1 can be around for at least a day; however, culturing cells in itself may have profound consequences for a protein’s lifetime. For our experiments, we used human erythrocytes as a source of hENT1. hENT1 is strongly expressed on erythrocytes that have an average lifespan of 120 days [38], suggesting that hENT1 degradation can be very slow. Hence, a long RT may make sense for hENT1 inhibitors, at the same time suggesting that in the current series of compounds RT may not be long enough for durable clinical efficacy as the longest RT measured (compound **28**) was 87 min. In an earlier study, we identified a draflazine analog with an RT of 628 min, while dilazep had an RT of 44 min [22]. As the compounds discussed in the present study were not brought to clinical trials, we can only speculate on the translational aspects of the above lines of thought. Draflazine, a closely related compound with an RT of 88 min under the same experimental protocol [22], has undergone extensive clinical trials to achieve cardioprotection. It was noted that high levels of target occupancy also induced on-target side effects in patients such as bronchospasms, while lower levels of target engagement (*e.g.*, 30%) did not cause these unwanted effects [16, 39, 40]. Therefore, as a cautionary prediction, long RT in hENT1 inhibitors should be aimed at achieving extended rather than high target occupancy.

Zooming in on the characteristics of the compounds in more detail, we learned that the R substituents may be responsible for long lasting binding, *i.e.*, RT, and hence a longer pharmacological effect of the inhibitors, whereas the composition of the main ring is responsible for a fast binding to the target (influencing *k*_*on*_ values) and an immediate effect (see also Fig. 1 and Tables 1, 2, 3, 4 and 5). These findings become of importance when the disease to be treated is taken into account. In the case of an acute disease, such as myocardial infarction, there is a need for a fast associating drug which immediately exerts its effect. On the other hand, for a chronic disease such as cancer, a slow dissociation is desired to maintain a longer physiological effect [41, 42]. If the RT exceeds the pharmacokinetic half-life, the drug can continue to have a sustained pharmacodynamic effect after plasma clearance. This, in principle, provides advantages like convenient dosing schedules for patients as well as preventing off-target toxicities [21, 43].

The data from the washout experiments (Fig. 2C) were in line with the data from the competition association assay. Additionally, this confirms that the RT is determined by binding to hENT1 and not rebinding, since all unbound inhibitor is taken out of the sample with washing. A possible occurrence of rebinding would also be substantiated by a correlation between lipophilicity (logD) and association rate constants, since lipophilic compounds are more likely to bind to the plasma membrane [44]. By binding to the plasma membrane, the drug concentration would locally increase and rise around the target, which facilitates the approach of the inhibitor to the transporter and subsequently prolongs the pharmacological effect [32, 45]. However, no such correlation between lipophilicity and association rate constants was observed, making membrane binding of these compounds not very probable.

Finally, the recent structure elucidation of hENT1 [46] provides further information on the binding sites of hENT1 inhibitors. Two reference and chemically diverse inhibitors, dilazep and 4-nitrobenzylthioinosine (NBTI) (Fig. 1), were individually co-crystallized showing that the two ligands occupy distinct locations that partially overlap. The new compounds in this study resemble dilazep more than NBTI, suggesting they may also occupy the extended channel region dilazep is residing in, facing the extracellular region.

## Conclusions

In this study, we tested a series of spirobenzo-oxazinepiperidinone derivatives designed as hENT1 inhibitors for their affinity and target binding kinetics by performing radioligand binding assays. Structure-kinetic relationships were examined in addition to structure-affinity relationships to define which functional groups are involved in binding to hENT1. It was found that bulkier substituents at the “right-hand” phenyl ring were well tolerated, suggesting a large binding pocket for hENT1. These substituents provided high affinity and a long RT when being hydrophobic and uncharged at physiological pH. Additionally, it was found that the compounds tested associate faster to the transporter when the polarity of the central scaffold is reduced. By and large, this study contributes to the development of inhibitors with high affinity and optimal binding kinetics at hENT1, and, more generally, paves the way for similar studies at other transport proteins.

### Supplementary Information

Below is the link to the electronic supplementary material.

Supplementary file1 (DOCX 168 KB)

## Data Availability

Data is contained within the article or supplementary material. Data not shown is available from the corresponding authors, upon reasonable request.
