# Supplementary material for: Kinetic profiling of novel spirobenzo-oxazinepiperidinone derivatives as equilibrative nucleoside transporter 1 inhibitors
Source: Purinergic Signal. 2023 Jul 10;20(2):193–205. doi: 10.1007/s11302-023-09948-9 (PMC10997566; doi:10.1007/s11302-023-09948-9)
Supplement: Supplementary file 1 — Supplementary file1 (DOCX 168 KB) [file 11302_2023_9948_MOESM1_ESM.docx]

Supplementary information for:

Synthesis and kinetic profiling of spirobenzo-oxazinepiperidinone derivatives as Equilibrative Nucleoside Transporter 1 inhibitors

Anna Vlachodimou^1^, Jara Bouma^1^, Michel De Cleyn^2^, Didier Berthelot^2^, Stefan Pype^2^, Jean-Paul Bosmans^2^, Herman van Vlijmen^1,2^, Berthold Wroblowski^2^, Laura H. Heitman^1^, Adriaan P. IJzerman^1^*.

^1^ Division of Drug Discovery and Safety, Leiden Academic Centre for Drug Research (LACDR), Leiden University, P.O. Box 9502, 2300 RA Leiden, The Netherlands; ^2^ Janssen Research and Development, Antwerpseweg 30, 2340 Beerse, Belgium

**Contents:**

- **Figure S1.** Structure of the ENT1 inhibitors and the subpart of the structure used for the pK_a_ (acid dissociation constant) and logD (distribution coefficient at pH 7.4) calculation (marked in blue)………………………………………………………………………….S3
- Table S1: pKa and logD (at pH 7.4) values determined for the phenyl ring including the R substituent of compounds 1 – 25………………………………………………………S4
- **Figure S2:** Correlation of affinity and kinetic parameters with physicochemical properties of inhibitors **1** – **25**.………………….…………………………………………………...S5

The physicochemical parameters of the R substituents were calculated using the Chemicalize platform from ChemAxon. The pK_a_ and logD values are displayed in Table S1 and correspond to the phenyl ring together with the R substituent (marked in blue in Supplementary Figure S1). The pK_a_ values were determined for the amine or oxygen on the phenyl *para*-position or the rest of amine and oxygens of the R substituents. We continued to the latter case, only when no ionization occurred in the first instance. The logD values were calculated for pH 7.4.

**Figure S1.** Structure of the ENT1 inhibitors and the subpart of the structure used for the pK_a_ and logD calculations (marked in blue).

Table S1: pK_a_ and logD (at pH 7.4) values calculated for the phenyl ring including the R substituent of compounds 1 – 25 (see Figure S1).

| **cmpd** | **pK_a_**  **(of N/O)** | **logD**  **(at pH 7.4)** |
| --- | --- | --- |
| **1** | 4.64^a^ | 1.14 |
| **2** | 4.68^a^ | 1.45 |
| **3** | 5.02^a^ | 2.08 |
| **4** | 14.38^a^ | 1.21 |
| **5** | 5.36^a^ | 1.86 |
| **6** | 5.04^a^ | 2.80 |
| **7** | 5.19^a^ | 3.11 |
| **8** | 3.93^a^  10.22^b^ | -1.54 |
| **9** | 5.00^a^ | 0.53 |
| **10** | 4.86^a^ | 2.2 |
| **11** | 1.43^a^ | 1.86 |
| **12** | 8.89^b^  -0.32^a^ | 0.05 |
| **13** | 7.95^b^  -0.88^a^ | 1.27 |
| **14** | 3.41^a^ | 2.83 |
| **15** | 4.86^a^ | 1.41 |
| **16** | 9.98^b^  3.63^a^ | -1.41 |
| **17** | 8.90^b^  3.31^a^ | -0.04 |
| **18** | 4.88^a^  -1.36^b^ | 0.70 |
| **19** | 4.7 ^a^ | 2.37 |
| **20** | N.D.^c^ | N.D. |
| **21** | No ionizable atoms found | 2.59 |
| **22** | No ionizable atoms found | 3.17 |
| **23** | 9.82^b^ | -0.90 |
| **24** | 8.6^b^ | 0.62 |
| **25** | -1.28^b^ | 1.07 |
| ^a^ Ionization of 4-N or 4-O. ^b^ Ionization of second substituted N or O. ^c^ N.D. not determined | | |


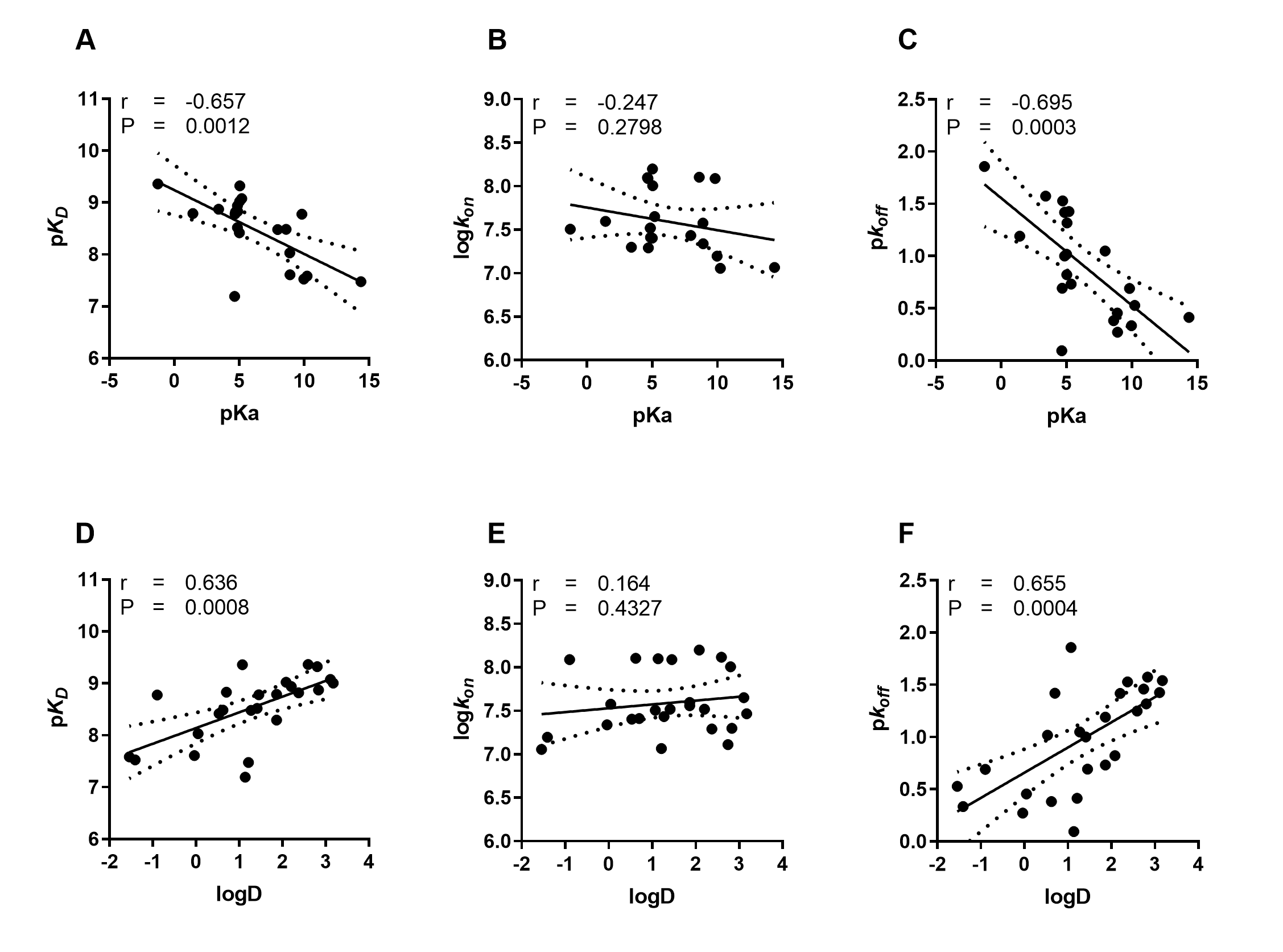


**Figure S2:** Correlation of affinity and kinetic parameters with physicochemical properties of inhibitors **1** – **25**.

The acid dissociation constant (pK_a­_) is correlated with affinity (p*K_D_*) (A); association rate constant (log*k_on_*) (B); and dissociation rate constant (p*k_off_*) (C). In addition, distribution-coefficient (logD) is correlated with affinity (p*K_D_*) (D); association rate constant (log*k_on_*) (E); and dissociation rate constant (p*k_off_*) (F). For all graphs, p*K_D_*, log*k_on_*, p*k_off_* are the values obtained for the binding of the whole molecule to the transporter. On the other hand, the acid dissociation constant (pKa) and the distribution-coefficient (logD) were calculated only for the R substituent. The solid line corresponds to the linear regression of the data, the dotted lines represent the 95% confidence intervals for regression. Data used in the plots are detailed in Tables 1-4.
